# A novel decision tree classification based on post-pruning with Bayes minimum risk

**DOI:** 10.1371/journal.pone.0194168

**Published:** 2018-04-04

**Authors:** Ahmed Mohamed Ahmed, Ahmet Rizaner, Ali Hakan Ulusoy

**Affiliations:** 1 College of Computer Science, University of Bahri, Khartoum, Sudan; 2 Department of Mathematics, Faculty of Arts & Sciences, Eastern Mediterranean University, Gazimağusa / North Cyprus, TURKEY; 3 Department of Information Technology, School of Computing and Technology, Eastern Mediterranean University, Gazimağusa / North Cyprus, TURKEY; Southwest University, CHINA

## Abstract

Pruning is applied in order to combat over-fitting problem where the tree is pruned back with the goal of identifying decision tree with the lowest error rate on previously unobserved instances, breaking ties in favour of smaller trees with high accuracy. In this paper, pruning with Bayes minimum risk is introduced for estimating the risk-rate. This method proceeds in a bottom-up fashion converting a parent node of a subtree to a leaf node if the estimated risk-rate of the parent node for that subtree is less than the risk-rates of its leaf. This paper proposes a post-pruning method that considers various evaluation standards such as attribute selection, accuracy, tree complexity, and time taken to prune the tree, precision/recall scores, TP/FN rates and area under ROC. The experimental results show that the proposed method produces better classification accuracy and its complexity is not much different than the complexities of reduced-error pruning and minimum-error pruning approaches. The experiments also demonstrate that the proposed method shows satisfactory performance in terms of precision score, recall score, TP rate, FP rate and area under ROC.

## Introduction

Decision tree is one of the most powerful and efficient techniques in data mining which has been widely used by researchers [1–3]. Compared to the other classification techniques the decision tree is faster and provides better accuracy. During the data classification process, some branches of the decision tree may contain noise or outliers in the training data and these results in a complex tree which is difficult to understand. Therefore, pruning techniques are applied in order to remove those unwanted branches with the aim of improving the accuracy, also removing non-productive parts of the tree results in less complex tree with small size [4–6].

There are two main pruning approaches: post-pruning and pre-pruning approaches. Post-pruning is implemented after the tree is grown. In practice, post-pruning methods have better performances than pre-pruning [7]. In pre-pruning, pruning is implemented during the tree building process and tries to stop the process when over-fitting is encountered. Hence, it prevents the generation of non-significant branches but suffers from horizon effect [8]. Pre-pruning method navigates the tree in a top-down approach while post-pruning navigates the tree in a bottom-up approach. Nevertheless, in term of simplification and complexity post-pruning algorithm is more robust since it has access to the full tree.

In the past decades, several post-pruning algorithms have been introduced such as reduced-error pruning, error-complexity pruning, minimum-error pruning, and cost-based pruning. Most of the pruning methods such as reduced-error pruning and minimum-error pruning traverse the decision tree in bottom-up order estimating the misclassification errors for each node to reduce the tree size and to avoid the over-fitting problem.

In this paper, we adopt post-pruning approach to combat the over-fitting problem that rises during data classification process and leads to a complex tree with large size and difficult to understand. To avoid this obstacle a new post-pruning method called Pruning with Bayes Minimum Risk (PBMR) is introduced in order to achieve high accuracy with reduced tree size. While post-pruning algorithms estimate the misclassification errors at each decision node, PBMR method estimates the risk-rate of a node and its leaf and then propagates this error up the tree instead of estimating the misclassification errors. If the parent node has a lower risk-rate than its leaf, the parent node is converted to a leaf node, otherwise, the parent node is retained. Several experiments are conducted to investigate the effectiveness of proposed PBMR method and its results are compared with the results of reduced-error pruning and minimum-error pruning approaches.

## Research issue

A decision tree is a flowchart-like tree structure, where each internal node denotes a test on an attribute, each branch represents an outcome of the test, and each leaf node holds a class label. Decision trees suffer from over-fitting problem that appears during data classification process and sometimes produce a tree that is large in size with unwanted branches. Pruning methods are introduced to combat this problem by removing the non-productive and meaningless branches to avoid the unnecessary tree complexity.

## Motivation

The advance progresses in information technologies result in a large amount of data that needs to be analysed and managed to gain useful information knowledge to predict future behaviour. Several types of research that the details will be discussed in Related Works section, have been conducted in the literature to store and manipulate this valuable data for further decision making. Although, decision tree is one of the most widely used data mining methods, it may provide very large trees in size. To overcome this problem several approaches such as pruning methods are introduced for optimizing the computational efficiency of the tree with high accuracy.

## Contribution

The contributions of this paper are the following:

- The paper indicates the importance of employing attribute evaluator methods to select the attributes with high impact on the dataset that provide more contribution to the accuracy.
- A new post-pruning method named as PBMR is introduced to overcome over-fitting problem and also to improve the accuracy performance.

## Related works

Several post-pruning algorithms for decision trees such as reduced-error pruning, pessimistic pruning, error-based pruning, cost-complexity pruning and minimum-error pruning have been introduced in the literature [9–11]. Each of these algorithms attempted to produce simple tree structure with high accuracy. Furthermore, post-pruning algorithms estimated misclassification errors at each decision node and propagated this error up the tree. The authors in [12] conducted a research which compared several pruning methods for error minimization. However, another research deduced that when error minimization was the evaluation criterion, most pruning algorithms resulted in trees that were larger than necessary [13]. Although the research in [14] performed an empirical comparison for five pruning methods, the experiment results showed that the methods such as critical-value pruning, error complexity pruning and reduced-error pruning outperformed the pessimistic-error pruning and minimum-error pruning in terms of the tree size and accuracy. Authors in [15] studied reduced-error pruning in different variants that were adding a new perspective to its algorithmic properties, analysing the algorithm with less assumption compared to previous analyses methods, and emptying subtrees in the analyses process. An experimental study for cost complexity pruning and C4.5’s error-based pruning that concentrated on pruning with loss minimization and probability estimation instead of error minimization was conducted in [16]. The study revealed that when the probability was estimated by Laplace correction at leaves level, all pruning methods were improved [16]. Furthermore, the study about error based pruning algorithm clarified that varying the certainty factors resulted in a smaller tree [17]. Therefore, error-based pruning produced applicable tree size with good accuracy compared to reduced-error pruning. Reduced-error pruning method in decision tree was also analysed in [18]. This study investigated the influence of pruning on the accuracy and tree size. The results showed that the produced tree was with small size and high accuracy. Post-pruning decision tree algorithm that was based on C5.0 decision tree algorithm and Bayesian posterior theory was introduced in [19]. The proposed method outperformed the original C5.0 decision tree algorithm and revealed that using Bayesian posterior theory as an enhancer for C5.0 classifier resulted in less memory and less classification time to search and build the rules.

## Bayes minimum risk

As defined in [20, 21], Bayes minimum risk classifier is a decision model based on quantifying trade-offs between various decisions using probabilities and the costs that accompany such decisions. The method suggested in this research considers a post-pruning approach that estimates the risk-rate for the parent node of the subtree and its leaves. The risk associated for each node *k* is computed as following:

$$R_{k}\left(a_{i}|x\right)=\sum_{j=1,j\neq i}^{T_{c}}L_{k}\left(a_{i}|C_{j}\right)p_{k}\left(C_{j}|x\right)$$ (1)

where *L*_*k*_(*a*_*i*_|*C*_*j*_) and *p*_*k*_(*C*_*j*_|*x*) are the loss function when an example is predicted in class *C*_*j*_ while true class is *C*_*i*_ and the estimated probability of an example belonging to *C*_*j*_, respectively and *T*_*c*_ is the total number of classes. The total risk of the leaves can be calculated as:

$$R_{l}=\sum_{m=1}^{T_{l}}R_{m}^{}\left(a_{i}|x\right)$$ (2)

where *T*_*l*_ is the total number of leaves under the subtree.

## Proposed algorithm

**Fig 1:**
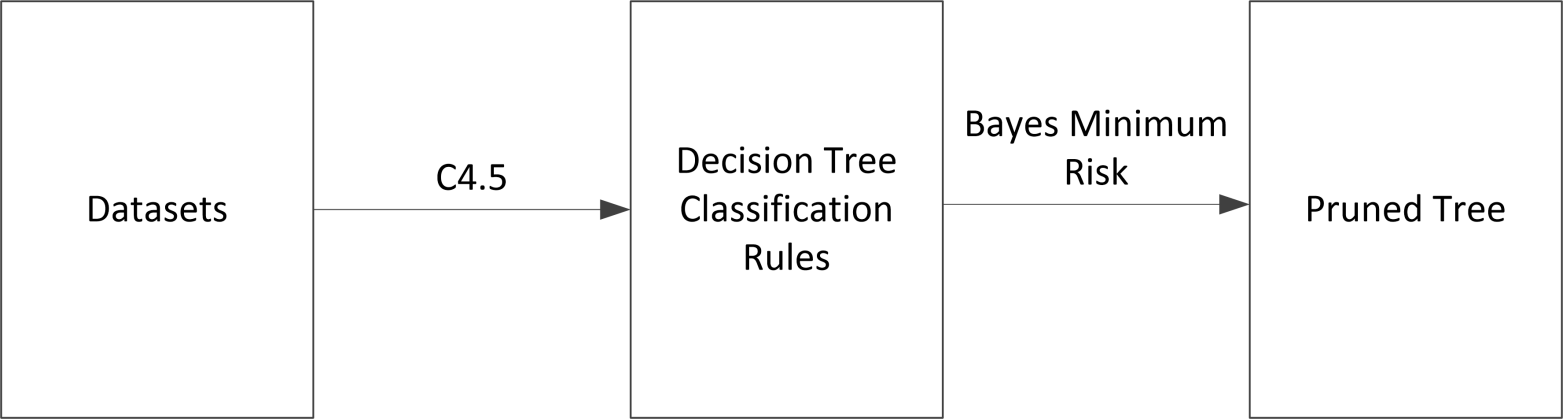
The principle of decision tree post-pruning algorithm based on Bayes minimum risk.

**Fig 2:**
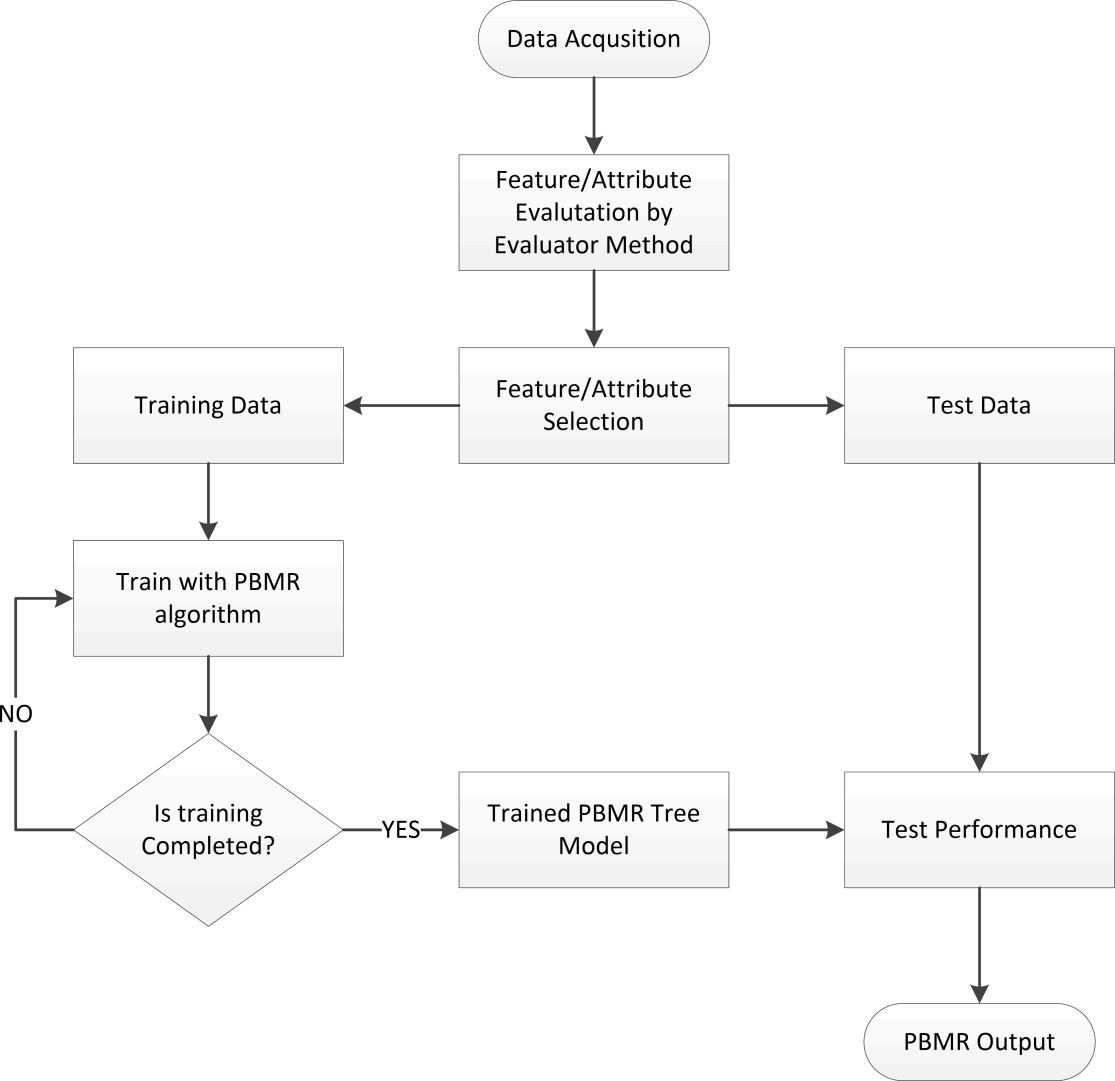
Proposed PBMR method flowchart.

In the proposed algorithm, a decision tree algorithm is used to build and initiate a tree model. Then linear regression method is applied to build models on leaves level of the tree. The proposed modified decision tree algorithm is implemented recursively with the following sequence until the tree is formed. Proposed algorithm adopts a post-pruning bottom-up method for C4.5 decision tree algorithm using Bayes minimum error method that estimates risk-rates instead of estimating the misclassification error as illustrated in Fig 1. Moreover, the flowchart in Fig 2 indicates the structure of the proposed algorithm and way followed to proceed.

**Fig 3:**
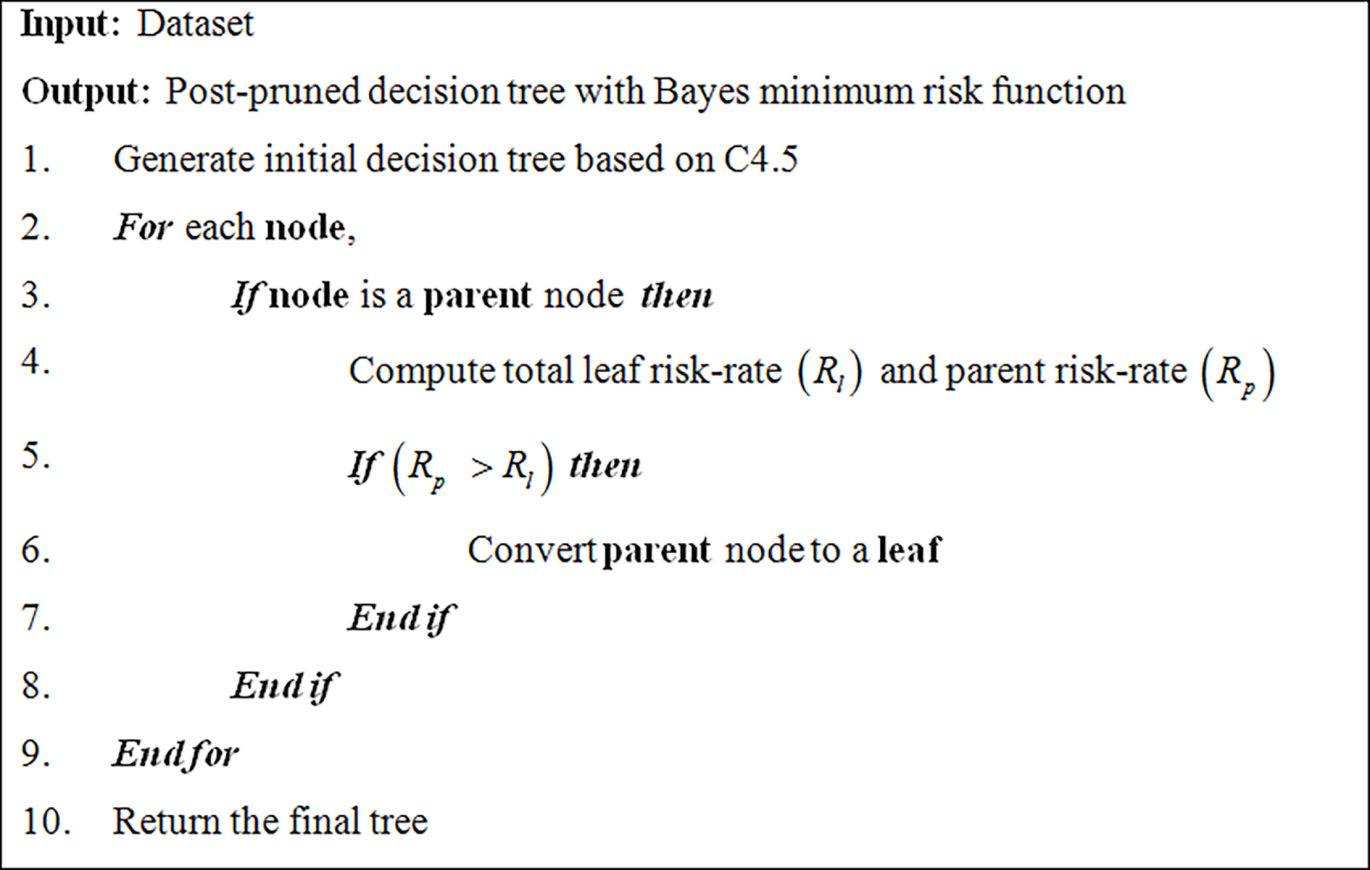
Proposed decision tree classification algorithm based on post-pruning with Bayes minimum risk.

After the decision tree is built, the proposed algorithm given in Fig 3 computes the risk-rates of the parent node of the subtree (*R*_*p*_) and the leaf nodes (*R*_*l*_) as in (1) and (2), respectively. The parent node is converted to a leaf node if the risk-rate of the parent is less than the total risk-rate of its leaves (*R*_*p*_ < *R*_*l*_), otherwise, the parent node is retained. The process is repeated for all parents of leaves until the tree is optimized. To clarify our notation, we illustrate the new method through a simple example. A simple decision tree example is given in Table 1.

**Table 1:** Example of a simple decision tree.

*a*	*b*	*c*	*class*
1	1	1	Yes
0	1	1	No
1	1	0	No
1	0	0	No
0	0	0	Yes

**Fig 4:**
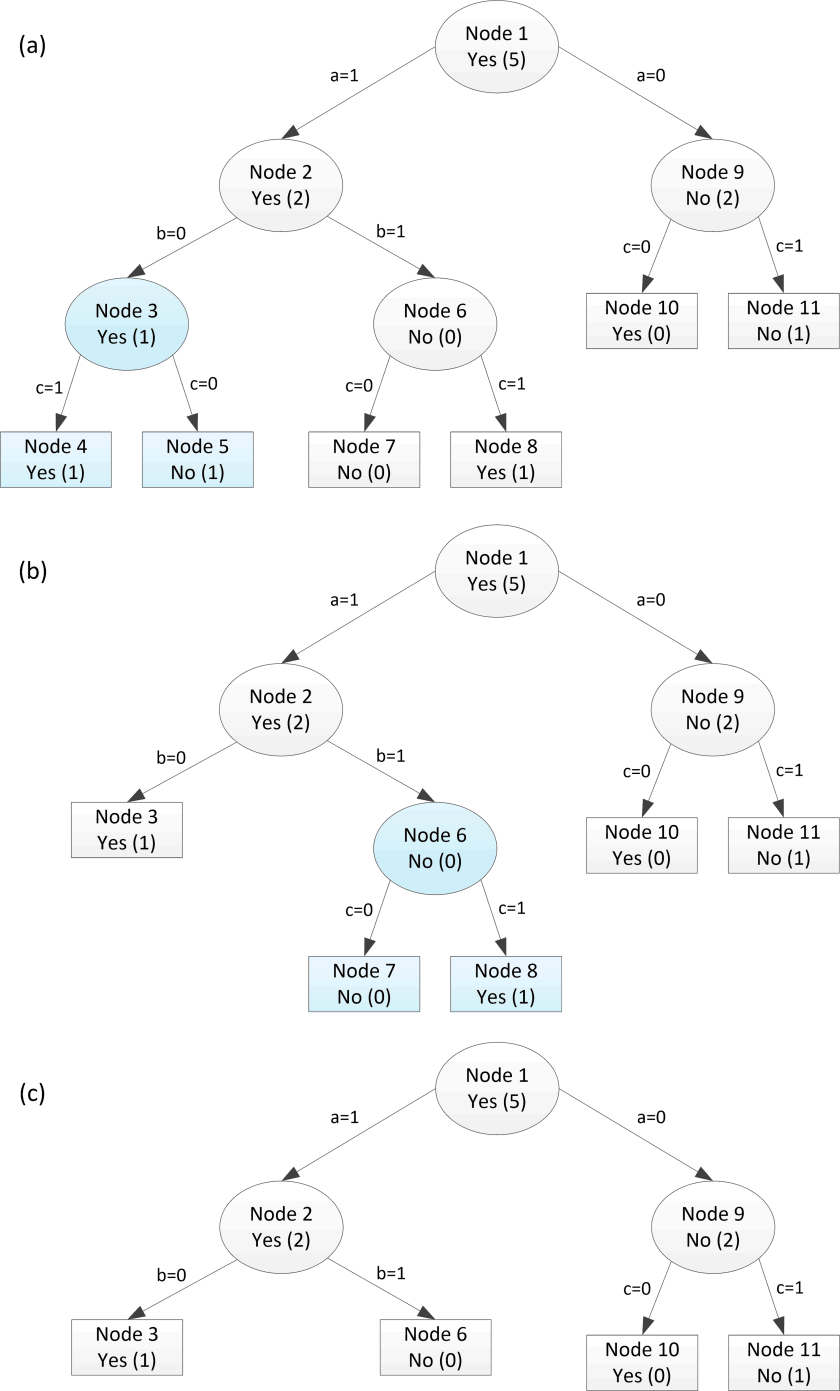
A simple decision tree example for PBMR method.

Fig 4 shows how the newly introduced method is applied to perform pruning operation on the given decision tree. The proposed method traverses the tree in a bottom-up fashion converting a node to a leaf if the risk-rate of the leaves is greater than the risk-rate of the node. To perform this task, the pruning method traverses the tree from left to right in bottom-up order. So that, in the first step the pruning method starts from the most left branch which is node 3 in our case as shown in Fig 4(A). Because the risk-rate of subtrees of node 3 (2) exceeds node 3 risk-rate (1), these subtrees are removed and node 3 becomes a leaf node given as in Fig 4(B). In the second step, the pruning method traverses nodes 6 converting it to a leaf since the risk-rate of its subtrees (1) is greater than the risk-rate of node 6 itself (0) as shown in Fig 4(C). Then in step three, node 2 is traversed after both of its successors are removed since the subtrees of node 2 has lower risk-rate (1) than node 2 itself (2), the subtrees are retained. In the last step, the risk-rates of the subtrees attached to node 0 (1) is less than the risk-rate of node 9 itself (2), so that the subtrees of node 9 are also retained for the same reason.

## Experimental results and discussions

To compare the proposed PBMR method with two other post-pruning algorithms namely, Reduced-Error Pruning (REP) and Minimum-Error Pruning (MEP), five different datasets, Zoo, Iris, Diabetes, Labour, and Blogger have been utilized [22]. Table 2 presents the number of instances, the number of classes, and the number of attributes for the datasets.

**Table 2:** Datasets description.

*Datasets*	*Number of Instances*	*Number of Attributes*	*Number of Classes*
Zoo	101	17	7
Iris	150	4	3
Diabetes	768	8	2
Labour	57	16	2
Blogger	100	6	2

Experiments are conducted by using java eclipse combined with Weka. It is known that attribute evaluator techniques can be applied to select the attributes that have the greatest impact on the dataset. Removing the worst ranked attributes that have lower importance on the dataset usually increases the accuracy of the algorithms [14]. In this context, Weka’s attribute evaluator techniques namely One Rule (OneR) and Information Gain (InfoGainAttributeEval) are employed to select the attributes with high impact on the datasets and remove the worst attributes that are shown in Table 3.

**Table 3:** Attributes removed by OneR and InfoGainAttributeEval attribute evaluators.

*Datasets*	*OneR*	*InfoGainAttributeEval*
Zoo	predator, catsize, domestic, venomous	predator, catsize, domestic, venomous,
Iris	sepalwidth, petallength	sepalwidth, sepallength
Diabetes	mass, pedi, skin	pres, pedi, skin, preg
Labour	wage2.wage, shift_diff, dur, hours.hrs, wage3.wage	stby_pay, dur, educ_allw.boolean, bereavement, boolean, wage3.wage
Blogger	lmt, lpss	lmt, lpss

After the worst attributes are removed with the attribute evaluators, the accuracies obtained by PBMR with 10-fold cross validation and by dividing the datasets into two sets as training and test are compared in Table 4. For 10-fold cross validation, datasets are partitioned into 10 subsets of equal size and each subset is employed for testing and the rest for training. Additionally, the same datasets are divided into two sets as training and test sets. 60% of each dataset is randomly selected as training set and 40% as testing set. The results show that PBMR with 10-fold OneR attribute evaluator achieves better accuracies for Zoo, Iris, Diabetes, and Labour datasets. Since both attribute evaluators removes the same attributes of Blogger dataset as shown in Table 4, the accuracy of PBMR with the both evaluators are equal. It is also noticed from the results that when 10-fold cross validation is used, better performance is obtained as compared to the case when datasets are divided into two as training and testing sets. Since 10-fold cross validation method with OneR attribute evaluator shows better performance, OneR attribute evaluator with 10-fold cross validation method is employed for the rest of the experiments.

**Table 4:** Accuracy of PBMR with OneR and InfoGainAttributeEval attribute evaluators.

*Datasets*	*Accuracy (%)*		
	*60% training*, *40% testing*, *OneR*	*10-fold*, *OneR*	*10-fold*, *InfoGainAttributeEval*
Zoo	85	88	86
Iris	95	97	95
Diabetes	73	76	73
Labour	74	75	74
Blogger	74	76	76

Table 5 shows the accuracy and the tree complexity in terms of tree size as the total number of nodes and leaves for PBMR, REP and MEP approaches. The results are also compared with the original un-pruned C4.5 decision tree algorithm (DT-C4.5) to illustrate the effect of pruning. For all the datasets, the proposed PBMR method produces better accuracies as 88%, 97%, 76%, 75% and 76% for Zoo, Iris, Diabetes, Labour and Blogger datasets respectively. In terms of complexity, PBMR produces smaller tree than REP and MEP for Iris dataset with seven nodes and four leaves. On the other hand, although PBMR produces greater tree than REP and MEP for Zoo, Labour and Blogger datasets, its performance in terms of accuracy is higher than the other methods. As seen from the results, besides of having better accuracy performance, all pruning-based approaches produce smaller tree sizes as compared to DT-C4.5.

**Table 5:** Accuracy and tree size for PBMR, REP and MEP.

*Datasets*	*Algorithms*	*Accuracy (%)*	*Number of Nodes*	*Number of Leaves*
Zoo	DT-C4.5	86	21	13
	PBMR	88	20	12
	REP	86	18	11
	MEP	76	12	8
Iris	DT-C4.5	93	13	7
	PBMR	97	7	4
	REP	95	9	5
	MEP	94	9	5
Diabetes	DT-C4.5	73	43	22
	PBMR	76	25	13
	REP	74	41	21
	MEP	74	15	8
Labour	DT-C4.5	72	47	39
	PBMR	75	45	37
	REP	65	9	7
	MEP	73	18	17
Blogger	DT-C4.5	73	43	28
	PBMR	76	36	25
	REP	72	15	11
	MEP	75	12	9

Moreover, the time taken by each pruning method to prune the tree is also considered. The experiment is repeated 100 times on a personal computer running on Windows 7 (64 bits) operating system with 2.55 GHz Dual-Core CPU and 4 GB RAM. The average results of the experiments are presented in Table 6. While PBMR method takes less time than REP and MEP methods to prune the tree in Zoo and Labour datasets, its pruning time is very close to the other pruning methods that has the shortest time for the remaining datasets.

**Table 6:** Time taken by PBMR, REP, and MEP to perform the pruning process.

*Datasets*	*Algorithms*	*Average Time (second)*
Zoo	PBMR	2.861
	REP	4.619
	MEP	3.212
Iris	PBMR	3.042
	REP	4.287
	MEP	2.887
Diabetes	PBMR	4.176
	REP	3.786
	MEP	5.475
Labour	PBMR	3.206
	REP	4.433
	MEP	4.124
Blogger	PBMR	3.185
	REP	3.073
	MEP	3.768

The next experiment includes the weighted average of precision and recall scores evaluations of the proposed method, REP, and MEP in Table 7. The precision and recall scores presented in Table 7 are compared in Tables 8 and 9. Score zero (0) represents worse algorithm and score one (1) represents better algorithm, whereas equal sign (=) represent equality [15]. As shown in Table 8, the precision of the proposed method is better than the precisions of REP, MEP, and DT-C4.5 with a score of three. On the other hand, the comparison of recall scores given in Table 9 shows that the proposed method is better than REP, MEP, and DT-C4.5 with a score of five.

**Table 7:** Precision and recall scores for PBMR, REP, and MEP.

*Datasets*	*Algorithms*	*Precision (%)*	*Recall* *(%)*
Zoo	DT-C4.5	85	86
	PBMR	87	88
	REP	86	86
	MEP	75	76
Iris	DT-C4.5	93	93
	PBMR	95	97
	REP	92	95
	MEP	94	94
Diabetes	DT-C4.5	73	73
	PBMR	75	76
	REP	73	74
	MEP	75	74
Labour	DT-C4.5	72	73
	PBMR	74	75
	REP	64	66
	MEP	73	73
Blogger	DT-C4.5	72	73
	PBMR	75	76
	REP	72	72
	MEP	75	75

**Table 8:** Precision scores of PBMR, REP, and MEP.

*Algorithms*	*Scores*					*Total Wins*
	Zoo	Iris	Diabetes	Labour	Blogger	
DT-C4.5	0	0	0	0	0	0
PBMR	1	1	=	1	=	3
REP	0	0	0	0	0	0
MEP	0	0	=	0	=	0

**Table 9:** Recall scores of PBMR, REP, and MEP.

*Algorithms*	*Scores*					*Total Wins*
	Zoo	Iris	Diabetes	Labour	Blogger	
DT-C4.5	0	0	0	0	0	0
PBMR	1	1	1	1	1	5
REP	0	0	0	0	0	0
MEP	0	0	0	0	0	0

The weighted averages of True Positive (TP) rate, False Positive (FP) rate and area under Receiver Operating Characteristic (ROC) curve are also considered to measure the performance of the pruning methods as in Table 10. The proposed method produces the highest TP rate in all datasets. The proposed PBMR method produces lowest FP rates for Zoo, Labour, and Blogger datasets. Moreover, the proposed PBMR method produces highest scores in terms of the area under ROC for all datasets.

**Table 10:** TP rate, FP rate, and area under ROC for PBMR, REP, and MEP methods.

*Datasets*	*Algorithms*	*TP Rate (%)*	*FP Rate (%)*	*Area under ROC (%)*
Zoo	DT-C4.5	86	3	92
	PBMR	88	1	94
	REP	86	2	93
	MEP	76	5	90
Iris	DT-C4.5	93	3	95
	PBMR	97	2	98
	REP	95	2	97
	MEP	94	3	97
Diabetes	DT-C4.5	73	34	77
	PBMR	76	29	83
	REP	74	29	78
	MEP	74	28	78
Labour	DT-C4.5	72	36	72
	PBMR	75	33	75
	REP	65	54	55
	MEP	73	39	72
Blogger	DT-C4.5	73	39	73
	PBMR	76	37	79
	REP	72	44	71
	MEP	75	41	73

## Conclusion

This paper introduces a new post-pruning method based on Bayes minimum risk. The efficiency of the proposed method in terms of attribute selection, accuracy, complexity, pruning time, precision score, recall score, TP rate, FP rate and area under ROC is compared to REP and MEP post-pruning methods by using five different datasets. The experimental results show that the proposed method produces better classification accuracy than REP and MEP in all test datasets while it does not create additional complexity than REP and MEP. The results also show that the proposed method yields satisfactory performance in terms of precision score, recall score, TP rate, FP rate and area under ROC compared to both REP and MEP approaches.

## Future work

The proposed algorithm adopts a post-pruning bottom-up method for C4.5 decision tree algorithm. As future works, the proposed PBMR method can be applied on C5.0 decision tree classifier and can be also modified for other tree base classifiers such as best first tree and random forest.
